# Integrated Strategies for Early Diagnosis and Prevention of Oral Diseases in Asia Pacific

**DOI:** 10.3390/ijerph22111737

**Published:** 2025-11-17

**Authors:** Liang Lin Seow, Michael Antonio F. Mendoza, Fatimah Maria Tadjoedin, Sheng-Wei Feng, Pong Pongprueksa, Linh Van Truong, Leo Gerald R. de Castro, Yun Yee Amber Lee, Vandana Garg, Melissa Adiatman, Loc Giang Do

**Affiliations:** 1 School of Dentistry, IMU University, Kuala Lumpur 57000, Malaysia; lianglin_seow@imu.edu.my; 2 Department of Community Dentistry, College of Dentistry, University of the Philippines, Manila 1000, Philippines; mfmendoza3@up.edu.ph; 3 Department of Periodontology, Faculty of Dentistry, Universitas Indonesia, Jakarta 10430, Indonesia; fatimah.tadjoedin@ui.ac.id; 4 School of Dentistry, College of Oral Medicine, Taipei Medical University, Taipei City 11031, Taiwan; shengwei@tmu.edu.tw; 5 Department of Operative Dentistry and Endodontics, Faculty of Dentistry, Mahidol University, Bangkok 10400, Thailand; pong.pon@mahidol.edu; 6 Private Dental Specialist, Ho Chi Minh City 700000, Vietnam; nhasilinh@gmail.com; 7 College of Dentistry, National University, Asian Center for the Dental Specialties, Manila 1008, Philippines; oralimplants@acds.com.ph; 8 Haleon Malaysia Sdn. Bhd., Ampang 68000, Malaysia; amber.x.lee@haleon.com; 9 Haleon Singapore Pte Ltd., Singapore 139234, Singapore; 10 Department of Dental Public Health and Preventive Dentistry, Faculty of Dentistry, Universitas Indonesia, Jakarta 10430, Indonesia; melissa31@ui.ac.id; 11 School of Dentistry, Faculty of Health, Medicine, and Behavioural Sciences, The University of Queensland, Brisbane, QLD 4006, Australia; l.do@uq.edu.au

**Keywords:** oral diseases, preventive strategies, systemic health, noncommunicable diseases, public health strategies, interprofessional collaboration

## Abstract

Oral diseases pose a significant global public health challenge, affecting 3.5 billion people and surpassing the prevalence of major noncommunicable diseases. There is a growing burden of oral diseases, including dental caries, periodontal diseases, and dentine hypersensitivity (DH) from several countries in Southeast Asia including Malaysia, the Philippines, Vietnam, Thailand, Indonesia, and Taiwan. The impact of oral diseases on quality of life and their association with systemic health emphasize the need for preventive strategies and early interventions. The objectives of the Oral Health Steering Committee were to gain insights and a comprehensive picture of the oral disease burden in the Southeast Asian region including Malaysia, the Philippines, Vietnam, Thailand, Indonesia, and Taiwan, to highlight the importance of early prevention of oral health diseases, as well as the importance of identifying early symptoms of oral discomfort associated with oral diseases and to examine the correlation between oral diseases and systemic health. The committee included nine experts from the region with significant expertise in dental health. A steering committee of nine dental experts from seven Southeast Asian countries reviewed evidence on the burden of oral diseases, the prevention of oral discomfort in early diagnosis and its impact on systemic health. The association between oral health and systemic health was discussed by the group. The committee highlighted that there is a need for comprehensive public health strategies, including population-level preventive measures, professional evaluations, and awareness programs to improve oral health outcomes and reduce treatment costs. The role in interprofessional collaboration between dental professionals and medical professionals in achieving early identification of oral diseases and increases referrals to dental professionals at early stages of diseases was highlighted. These insights stress the importance of early diagnosis, prevention and targeted interventions to mitigate the impact of oral health issues and improve overall public health outcomes in the region.

## 1. Introduction

Oral diseases affect people of all ages across the globe and can significantly impact their quality of life (QoL). The prevalence of the two most common oral diseases (dental caries and periodontal diseases) surpasses the prevalence of the five most common noncommunicable diseases (NCDs) including cardiovascular disease, diabetes mellitus, chronic respiratory disease, cancer and mental health issues [1,2,3]. Half the world’s population suffers from oral diseases. Despite being preventable through improved oral hygiene and risk factor control, the prevention of oral diseases is often not prioritized. Dental treatment has largely focused on restorative and rehabilitative treatment. Low-income countries and rural areas have limited access to oral healthcare, and patients often consult a dental professional only for emergencies [1,2,4]. Furthermore, elderly individuals have a higher risk of oral diseases due to cognitive and functional decline, dietary limitations, reduced salivary flow, and polypharmacy [1,2]. Routine dental care is not a priority in most countries due to limited access to healthcare and lack of awareness [2,5].

Oral diseases occur across the life span, with an increase in severity reported with an increase in age. Even when oral diseases occur in early adulthood, they continue to impact an individual as they age, leading to reduced quality-adjusted life expectancy. Enhanced preventive measures, early detection and management of oral diseases can improve population health. However, comprehensive public health strategies are necessary to do this effectively [1].

Oral diseases and NCDs have common modifiable risk factors such as smoking, unhealthy diet, frequent snacking habits and alcohol consumption. Oral diseases have a close relation to systemic diseases, e.g., cardiovascular disease and diabetes mellitus. Therefore, managing oral health through prevention as well as curative treatment can have a beneficial impact on systemic health. Preventive measures can be implemented at the population level, through education, increasing public awareness, improving access to oral healthcare and fluoridation of water. Increased awareness may benefit individual-level preventive measures through improved brushing, interdental cleaning and seeking early professional help along with risk factor control. Routine professional evaluation and preventive measures as simple as appropriate brushing techniques, usage of fluoridated toothpaste and frequency of brushing can aid in preventing and slowing the progression of oral diseases [1,2,6]. This paper discusses data from the Southeast Asian region, including Malaysia, the Philippines, Vietnam, Thailand, Indonesia, and Taiwan.

### 1.1. Burden of Oral Diseases in Southeast Asia

According to estimates by the World Health Organization, 3.5 billion people suffer from an oral disease [7]. Globally, 2.5 billion individuals are affected by untreated dental caries. The prevalence of oral diseases is on the rise globally, surpassing the rate of population growth [8]. One disability-adjusted life-years (DALY) is considered to be one year of “healthy life” lost due to either premature mortality or disability [9]. DALY rates attributable to oral disorders are reported in Table 1 [8,10]. Years lived with disability for tooth loss (YLDs-T) was 1445 per 100,000 in Taiwan, with the highest value of YLDs-T noted for the first molar [11].

Dental caries is a chronic disease affecting people of all ages. Table 1 describes the burden of dental caries in various countries [12,13,14,15,16]. Vietnam reports a significant burden of dental caries, with a higher prevalence reported in obese individuals (80.2% vs. 55.6% for normal weight individuals) [12]. In Thailand, lower education was a risk factor for dental caries [13]. National Health Survey data from Indonesia reported a high prevalence of caries, and risk factors included residence in rural areas, low formal education, consumption of sweets and sugary beverages at least once a day, lack of regular tooth brushing, and smoking [14]. In Malaysia, the burden of dental caries was high (85%) in 2020 but had decreased from that in 1990 (94.6%), according to the National Oral Health Survey [15]. The Philippines is reported to have a high prevalence of dental caries (87.4%) with a low rate of treatment [16].

Periodontal disease (PD) is a leading cause of tooth loss and impacts systemic health as well [15,17]. Table 1 describes the burden of PD in various countries [15,17,18,19,20,21]. In Vietnam, the prevalence of PD among adults was reported to be 64.9% with a higher prevalence among individuals aged over 65 years [18]. The National Oral Health Survey of Thailand reported a prevalence of 25.9–36.3% [19]. Indonesia has a high burden of PD, particularly among individuals with diabetes mellitus and older adults [20,21]. Severe periodontal destruction is reported for 33.3% of individuals in Indonesia, while 97.3% were reported to have periodontitis. The prevalence of severe periodontitis was higher among older individuals [21]. In Taiwan, 784 per 1000 outpatient visits were for PD [17]. The National Oral Health Survey of Malaysia reported a PD burden of >90% in the year 2020 [15].

Dentine hypersensitivity (DH) is a common oral condition with a reported prevalence up to 98%, substantially impacting oral health-related quality of life (HRQoL) [22]. Table 1 describes the burden of DH in various countries [23,24,25]. In Vietnam, the prevalence of DH was higher among those aged 40–49 years compared with 30–39 years (30% vs. 24%) [23]. In contrast, a study in Thailand reported a higher prevalence among females compared with males (70.5% vs. 29.5%) and those aged 30–39 years compared with 40–49 years (44% vs. 40%) [24]. In Taiwan, a study reported that 32% of patients had current cervical DH, while 12% reported a history of hypersensitive teeth [25].

**Table 1:** Burden of oral diseases and associated morbidity in various countries in Asia Pacific [8,10,11,12,13,14,15,16,17,18,19,20,21,23,24,25].

Country	Reported Disease Burden			Morbidity Associated with Oral Diseases
	Dental Caries	Periodontal Disease	Dentine Hypersensitivity	
Indonesia	88.8% ^†^ [14]	71% (periodontitis); 92% (DM individuals with periodontitis [20], and 33.3% (severe periodontal destruction) [21]	-	DALY: 282.8 [8]
Malaysia	85% ^†^ [15]	94.5% ^†^ [15]	-	DALY: 71,449; PD: 26,680; dental caries: 4125 [10]
Philippines	87.4% [16]	-	-	-
Taiwan	-	Use rate * of 38.6% [17]	32% (current cervical DH), 12% (history of DH) [25]	YLDs-T: 1445 per 100,000 [11]
Thailand	35.2% ^†^ [13]	Adults 25.9%, Elderly 36.3% [19]	Males 70.5%, Females 29.5% [24]	DALY: 333.3 [8]
Vietnam	53.1% ^†^ [12]	64.9% ^†^ [18]	20.4% ^†^ [23]	

DM: Diabetes mellitus; DH: Dentine hypersensitivity; PD: Periodontal disease; DALY: Disability-Adjusted Life-Years; YLDs-T: Years Lived with Disability for Tooth Loss. * Dental use rate data (calculated by dividing the number of patients in each age group by the total population of the same age group). ^†^ Prevalence among adults.

Oral health inequalities among low-income groups magnifies the burden of tooth and gum disease, leading to lower overall health and QoL. Studies indicate that improving oral health can lead to savings of up to US$ 7.4 billion in lifetime tooth decays costs in Indonesia. The corresponding savings in Thailand, Vietnam and the Philippines are US$ 1.7 billion, US$ 1.5 billion, and US$ 4.7 billion, respectively. Additionally, effective management of gum disease can lead to savings of US$ 1.5 billion in costs associated with Type-2 diabetes mellitus over 10 years in Indonesia. The corresponding savings for corresponding savings in Thailand, Vietnam and the Philippines are US$ 882 million, US$ 294 million, and US$ 9.7 billion, respectively [26,27,28,29].

### 1.2. Purpose of the Study

There is a substantial burden of oral diseases in Southeast Asia with a significant impact on QoL. Therefore, there is a need for increasing public awareness and early detection, which could translate to increased preventive and non-surgical management, and fewer surgical interventions, potentially lowering the cost of care. Towards this, a group of dental experts in the Southeast Asian Region convened to discuss the burden of oral health ailments and strategies to address these early detection and preventive care, with a focus on highlighting the importance of preventive measures in oral health. The premise was that addressing the most common oral health concerns with a focus on prevention, can lead to optimal health, QoL and aging.

## 2. Materials and Methods

The objectives of the Oral Health Steering Committee were to gain insights and a comprehensive picture of the oral disease burden in the Southeast Asian region including Malaysia, the Philippines, Vietnam, Thailand, Indonesia, and Taiwan, to highlight the importance of early prevention of oral health diseases, as well as the importance of identifying early symptoms of oral discomfort associated with oral diseases and to examine the correlation between oral diseases and systemic health.

The expert committee reviewed the evidence on the burden of oral health ailments and discussed the impact of oral discomfort, which could be a sign of other oral health issues such as DH, dental caries, and periodontal disease, and their effects on general health. The association between oral health and systemic health was discussed by the group. The key highlights of the discussion are presented in this manuscript.

### Expert Committee

Expert committee members were invited to participate based on their expertise in the field and the number of years of clinical experience (>15 years). The expert committee included nine members with six specialties from seven countries: Dr Seow Liang Lin (Malaysia; restorative dentistry), Dr Michael Antonio Mendoza (The Philippines; health policy, dental public health, and community dentistry), Dr Fatimah Maria Tadjoedin (Indonesia; periodontics), Dr Sheng-Wei Feng (Taiwan; prosthodontic dentistry), Dr Pong Pongprueksa (Thailand; restorative materials and technique), Dr Linh Van Truong (Vietnam; clinical general dentistry), Dr Leo Gerald R. de Castro (The Philippines; prosthodontic and implant dentistry), Dr Melissa Adiatman (Indonesia; geriatric dentistry and public health dentistry), and Dr Loc Giang Do (Australia; oral epidemiology, dental public health).

## 3. Oral Discomfort Can Be an Early Indicator of Other Oral Health Diseases

Oral discomfort is the discomfort that involves both hard and soft tissues in the oral cavity such as odontalgia, sensitivity, pain, bleeding gums, gum inflammation, and chewing difficulty. Hence, oral discomfort is not limited to tooth discomfort alone but also involves the gingiva and surrounding structures. The discomfort can arise due to, but not limited to, the oral conditions described in this section, i.e., dental caries, DH and periodontal disease. It is important to prevent and identify each condition in the early stages, so prophylactic and curative action can be taken to prevent complications and disease progression.

### 3.1. Dental Caries

**Figure 1:**
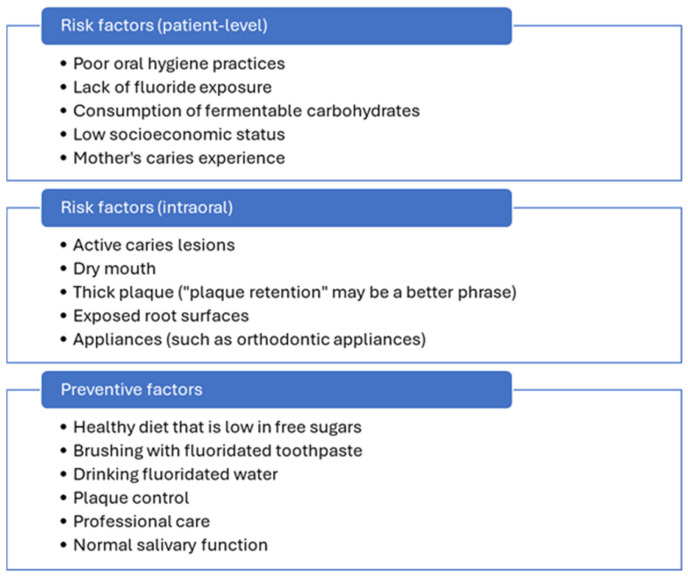
Risk factors and protective factors associated with dental caries [30,31,32].

Dental caries is the result of an unfavorable oral environment involving a shift in the acid production by the oral microbiome due to dietary and behavioral factors. Dietary factors include increased consumption of fermentable carbohydrates, particularly sucrose. Behavioral factors leading to dental caries include poor oral hygiene practices. Risk factors and preventive factors are described in Figure 1 [30,31,32]. Protective factors include maintaining normal salivary functions, healthy diet, proper brushing technique with fluoridated toothpaste, drinking fluoridated water and professional fluoride application and preventive sealants [30,33].

Diagnosis of caries requires assessment of risk factors and clinical examination. During examination, early-stage and late-stage caries lesions can be best identified on dry, plaque-free teeth after tooth cleaning. The severity of caries lesions is based on the clinical appearance of the tooth surfaces. An active lesion has one or more characteristics including being in a plaque stagnation area, whitish/yellowish in color, matte, has a rough tactile feeling on probing, visible cavitation or dentinal involvement, and may be associated with gingival bleeding. Initial or early caries lesions (white spot lesions) require control of progression, while moderate or extensive lesions require surgical intervention. Therefore, detection of dental caries at an early stage is crucial as it can prevent the need for surgical management [30,32]. It has been reported in the Malaysia National Oral Health Survey of Adults (NOHSA) of 2024 that the prevalence of caries is 85.1%, but extraction is required in 28.9% of patients and restorative care in 40.7% of patients [34]. The high rate of surgical management indicates that early detection is lacking. This underscores the need for greater awareness and timely professional evaluation of oral health.

If dental caries is detected and managed at an early stage, e.g., at the stage when it only involves the enamel, it can be managed conservatively with topical fluoride application and reinforcement of oral hygiene measures to promote remineralization of the early caries lesions. This can help prevent progression of disease, and prevent more invasive treatments, e.g., having to do restoration. Dentifrices with fluoride can help in remineralization and prevent further caries progression. Remineralization is the key to the early management of dental caries and can reverse the disease at an early stage. If caries is at an advanced stage, root canal treatment or tooth extraction may be required.

It is important to incorporate education about oral hygiene in schools and at the community level. Dental caries of deciduous teeth in children is largely ignored by parents due to the thought that “milk teeth” are replaced by permanent teeth. However, besides masticatory and speech function, deciduous teeth also play an important role as space maintenance is important. If deciduous molars are lost before the age of 6–7 years, then the first permanent molar may erupt in the wrong space, thus leaving less space for the permanent premolars. This may cause crowding, leading to aesthetic and functional problems. Ultimately, the child may require orthodontic treatment. Therefore, it is important to counsel the parents that deciduous teeth serve the purpose of maintaining space for the permanent teeth to erupt in the appropriate position.

The awareness of oral health among the public is not very high, and people feel that drilling/filling or extraction is the solution for caries; therefore, prevention is not given importance. Awareness programs are paramount in educating the public and raise appreciation on oral health by videos, online games, magazines and other digital solutions. Some of the adult population know that they need treatment, but they delay treatment for various reasons including dental anxiety, cost of treatment, etc. Extractions may be an acceptable course of action for many people to avoid more complex treatment. The cost of treatment is also a challenge as the number of people who can afford to undergo high treatment costs are lower. Thus, when early-stage intervention is not offered to the population, this may complicate the management strategy at a later stage.

### 3.2. Dentine Hypersensitivity

Dentine hypersensitivity (DH) can be described as a short, sharp pain when dentine is exposed to external stimuli (including thermal, vapor, tactile, osmotic, or chemical stimuli) not associated with any other dental disease or defect [35]. Processes that contribute to the development of DH include attrition and erosion of enamel, corrosion, abrasion, and abfraction. Exposure of the cervical and root dentine can result from periodontal tissue loss or gingival recession. Patient factors that contribute to DH include aggressive brushing [36]. Additionally, tooth wear may be a risk factor for DH, wherein up to 50% of patients with tooth wear are diagnosed with DH, compared to 12% of patients without tooth wear who are diagnosed with DH [10]. Enamel and dentinal wear at the cervical region and dentine wear at the cervical region are positively correlated with DH [37]. An increase in the severity of tooth wear measured using the Basic Erosive Wear Examination (BEWE) is correlated to increasing severity of DH on occlusal and incisal surfaces measured using the Cumulative Hypersensitivity Index (CHI) [38]. The increasing prevalence of tooth wear along with the consumption of erosive diets among younger populations has led to an increasing prevalence of DH. DH can be considered a clinical indication of active erosive tooth wear [39].

**Figure 2:**
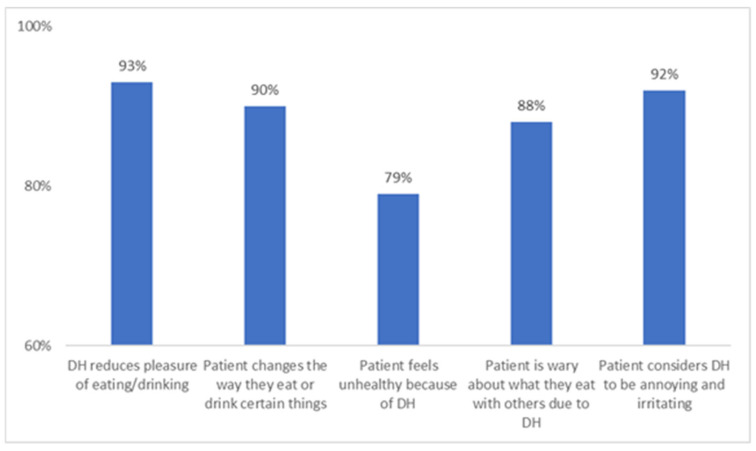
Impact of dentine hypersensitivity on quality-of-life using the Dentine Hypersensitivity Experience Questionnaire (DHEQ) [40].

Diagnosis of DH requires the exclusion of other diseases that may cause similar symptoms. Gingival recession leading to exposed dentine can point to suspected DH, which can be confirmed with tactile examination using a probe or an air blast test [36]. DH has a significant effect on QoL, as reported through evaluation using the Dentine Hypersensitivity Experience Questionnaire (DHEQ; Figure 2). DH is seen to impact daily functioning coping behaviors, as well as social and emotional impact [40,41].

Despite the significant impact of DH, it is reported that only 10–25% of patients seek treatment for the discomfort caused by consuming hot or cold foods [22]. Only 24.8% of participants with DH used desensitizing toothpaste [42].

It is important to differentiate between DH and sensitivity due to pulpal inflammation (such as after bleaching) which is usually transient and subsides within 48 to 72 h. Active ingredients such as Novamin (a calcium sodium phosphosilicate bioactive glass), Stannous Fluoride, and Potassium Nitrate, have been proven to be effective in the management of such cases. In addition, dentists must be educated on the appropriate product and active ingredient to use for DH and post-operative pulpal inflammation.

The management of DH requires long-term use of appropriate products. Patients have a certain level of expectation regarding the treatment, and counseling regarding long-term management is crucial. Dentists must counsel the patients on the correct use of the product and the duration of use so that effective relief can be achieved. There are different variants of products available in the market and the patient must be informed and educated regarding the active ingredients of the products and how the products work, to make informed decisions.

DH, though prevalent across adult populations, does show higher prevalence in certain groups. From a public health perspective, it is important to recognize patient groups that are at higher risk of developing DH (existence of enamel erosion and gingival recession, aggressive toothbrushing behavior) and educate them about DH. This would serve as a targeted public health intervention and help promote preventive behaviors. Age is another factor that needs to be considered in patient education. The older generation is habituated to rinse their mouth after brushing. However, the younger generation has seen messages of only spitting out after brushing, rather than rinsing out. Targeted educational campaigns should be utilized to achieve a change in oral hygiene habits.

### 3.3. Periodontal Diseases

**Figure 3:**
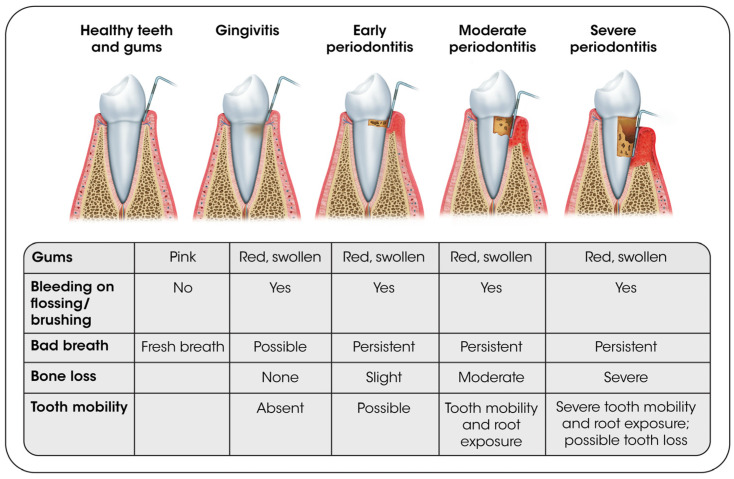
Progression of gingivitis to severe periodontitis and tooth loss [46].

Periodontal disease encompasses gingivitis and periodontitis, which involve inflammation of the gingiva, alveolar bone, and periodontal ligaments that surround the teeth [43]. Mild periodontitis is defined as ≥2 interproximal sites with attachment loss (AL) ≥ 3 mm, and ≥2 interproximal sites with probing depth (PD) ≥ 4 mm (not on same tooth) or one site with PD ≥ 5 mm. Moderate periodontitis is defined as ≥2 interproximal sites with AL ≥ 4 mm (not on same tooth), or ≥2 interproximal sites with PD ≥ 5 mm (not on same tooth), and severe periodontitis is defined as ≥2 interproximal sites with AL ≥ 6 mm (not on same tooth) and ≥1 interproximal site with PD ≥ 5 mm [44]. Gingivitis is the localized inflammation of the gingiva which occurs due to bacteria in dental plaque. It is defined as “10% or more of sites with positive bleeding on probing (full mouth bleeding on probing score [FMBS] ≥ 10%) and no clinical attachment loss” [43,45]. Figure 3 illustrates the progression of gingivitis to periodontitis and tooth loss [46].

Untreated gingivitis can lead to chronic periodontitis, wherein loss of bone and periodontal ligament occur, and periodontal “pockets” develop, ultimately leading to tooth loss. Periodontitis is defined as “interdental clinical attachment loss detectable at ≥2 non-adjacent teeth, or buccal or oral clinical attachment loss ≥3 mm with pocketing ≥3 mm detectable at ≥2 teeth.” Gingivitis is reversible with debridement followed by oral hygiene and follow-up professional preventive care, while periodontitis is largely irreversible. The management is complicated by the fact that the early stages of periodontal disease are painless, and it is only when the disease has progressed in severity that a diagnosis is made [43,45].

Periodontal disease is multifactorial in nature and results from the presence of pathogenic bacteria, the host inflammatory and immune responses, and other identified environmental and systemic risk factors [47]. Periodontitis and gingivitis are mainly initiated by dental plaque. Damage of the periodontal structure occurs by baleful byproducts and enzymes from periodontal bacteria, as well as microbial biofilm formation [47,48]. Age is a key risk factor associated with periodontal diseases. The prevalence of periodontal disease increases with age from adolescents to adults and the older population. It is reported that AL ≥ 6 mm and PD ≥ 5 mm occurs in 40.7% and 22.7% of people aged 65 years and older, respectively. The prevalence of periodontitis in this age group is reported to be 66% [49,50]. Other risk factors for periodontal diseases are listed in Table 2 [49,50,51,52,53,54,55,56]. Some of these are risk factors for NCDs as well, thus highlighting the association between oral health and systemic health.

The diagnosis of periodontal disease requires the assessment of periodontal probing depth, bleeding on probing, gingival recession, mucogingival deformity, furcation involvement, tooth mobility, biofilm index, and occlusal trauma. Radiography aids in identifying the extent of horizontal and vertical alveolar bone loss [47].

According to the data for patients with periodontal diseases in Indonesia, a majority of patients at diagnosis are at later stages (stage III: 57.6%; stage IV: 14%). This is most likely because periodontal health is not considered a priority [45]. Globally, 10–15% of the population is suffering from tooth loss due to periodontal disease [48]. Early symptoms of periodontal disease are often ignored, resulting in patients seeking periodontal therapy at an advanced stage of the disease. Successful prevention could reduce tooth loss and the need for restorative treatment to replace missing teeth. Subsequently, the cost of treatment would decrease due to a reduced need for dentures and dental implants, even in an aging population [51,52].

The correlation between diabetes and periodontal diseases has been well-documented and can be attributed to common risk factors. Patients with chronic periodontal diseases or with severe bone loss are encouraged to be investigated for diabetes. The management of periodontal diseases in patients with diabetes requires collaboration with medical practitioners to improve diabetic control. Diabetic control will help to improve the outcome of periodontal treatment and vice versa. This aspect requires educating patients on the correlation between these conditions. Knowledge of the correlation of disease should also be included, not only in dental medicine curricula, but in medical curricula as well. Interprofessional education should be made an integral part of curricula. The undergraduate curriculum needs to see more prominence of interprofessional learning. Physicians with knowledge of oral health conditions will be able to provide counseling as part of comprehensive health counseling. Imparting knowledge on oral health to physicians will also enable them to help patients identify early symptoms of oral diseases (which are often neglected by patients) and refer them to dental professionals for further care. This approach assumes greater importance in rural areas where dental care facilities may be limited, and where knowledge of oral diseases among the population is lacking.

There are several challenges faced in periodontal disease management. Patients do not prefer invasive surgery, and periodontal surgery can cause pain and hypersensitivity. Due to this, a number of patients do not return for continuous care. Periodontal patients must be made aware that treatment will be required for their lifetime to maintain periodontal health. Regular check-ups with a dentist/periodontist are necessary, and the interval will be determined by the staging and activity of the disease. Complete dental care is necessary, and some patients may need to undergo endodontic treatment or tooth extraction. Surgical management is considered the last step, and hence, patients should be motivated to maintain oral hygiene. Laypersons can recognize caries or cavities visually, but there is no visual clue for periodontal disease until the tooth becomes mobile, which is the advanced stage of periodontal disease. There is a need to increase awareness of periodontal diseases.

Tele-dentistry for follow-up visits of periodontal patients can be explored to maintain continuous care. This would be helpful for patients in remote areas as they may not be able to visit the specialist frequently for the maintenance phase. For such patients, scaling and polishing treatment can be performed at a local clinic, and specialist consultation could be carried out via tele-dentistry consultation. Tele-dentistry may require the use of an intraoral camera which is affordable and can be used by the patient during tele-dentistry consultation sessions. However, it may be a less practical option due to financial and technological barriers.

## 4. NCDs Are Associated with Oral Health Conditions

**Figure 4:**
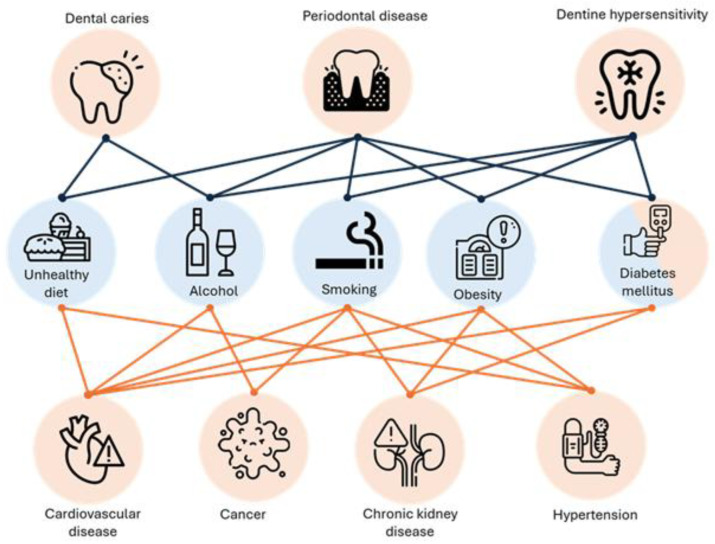
Association of common risk factors with oral diseases and noncommunicable diseases (NCDs) [45,47,48,51,52,53].

Oral health has been considered as the gateway to overall health. Poor oral health can lead to systemic complications. This can be attributed to common modifiable risk factors (Figure 4) [45,47,48,51,52,53]. Risk factor management for oral diseases would also reduce the risk of noncommunicable diseases (NCDs). However, oral health is not considered a priority in several countries, and the burden of certain oral diseases has not reduced in the past decades. While dentistry has made great advances in treatment of oral disease, prevention is lacking, likely due to inadequate funding and policy prioritization [2,5,6].

A meta-analysis reported that 28 NCDs were strongly associated with oral diseases [53]. These include diabetes mellitus, cardiovascular disease, obesity, cancer, asthma, inflammatory bowel disease, gastric helicobacter pylori infection, rheumatic diseases, neurodegenerative conditions, depression, etc. [53]. The severity of periodontitis is greater in diabetic patients compared with non-diabetic patients, with evidence of deeper pocket probing depth (mean difference [MD] 0.23 mm), elevated plaque index (MD 0.20 mm), and more teeth with bleeding on probing (MD 7.90) [57]. Diabetes patients may have up to a threefold higher risk of developing periodontal diseases than the general population and the risk depends on the level of glycemic control. The severity of periodontal diseases is 10 times higher for smokers with diabetes [58]. Diabetes increases the incidence or risk of progression of patients with periodontal diseases by 86% [59].

Periodontal disease is associated with decreased high-density lipoprotein cholesterol levels, while improvement in oral hygiene can reduce the risk of dyslipidemia [60]. A 50–150% higher odds of cardiovascular disease is noted for individuals with high numbers of decayed, missing and filled teeth (DMFT) scores of 14 or higher or with missing teeth. Missing teeth could be an indicator of chronic systemic inflammation and cumulative caries burden. In contrast, brushing and flossing was associated with 52% lower odds of cardiovascular disease. Tooth loss has also been associated with the occurrence of coronary artery disease and increased risk of stroke-related mortality. The chronic inflammation associated with periodontal disease may be the cause of the elevated cardiovascular risk. Furthermore, the accumulation of microbes in damaged oral tissues also increases the cardiovascular risk [61].

Given the association between NCDs and oral disease, and the spectrum of shared risk factors, a multidisciplinary approach could benefit not only oral health but NCDs as well. Dental professionals may be able to identify patients at risk of NCDs, and this could help identification in previously undiagnosed patients. A common risk factor approach could benefit oral and systemic health, and these targeted common risk factors (by dental and medical professionals) would impact a range of conditions and would thus be a cost-effective approach [62]. Oral health is largely ignored by medical professionals. Incorporation of this knowledge in the curricula for both dental and medical professionals could be of benefit. In addition, providing general and oral healthcare at public health facilities may reinforce the practice of risk factor management. Therefore, adopting a holistic and collaborative approach to the management of NCDs and oral diseases would reduce the public health burden.

## 5. The Importance of Prevention in Oral Health

Achieving oral health with ideal functioning and aesthetics requires effective self-care along with timely professional evaluation and intervention. Self-care involves consumption of a healthy diet, risk factor control (smoking and alcohol cessation), and tooth brushing with fluoridated toothpaste and timely check-ups. Dental professionals have the responsibility of monitoring oral health, identifying patients at risk of developing oral diseases and progression of the diseases, and providing appropriate treatment and intervention procedures.

Despite being preventable (Table 2 [49,50,51,52,53,54,55,56]), the prevalence of oral diseases remains high, and treatment seeking or diagnosis often happens at a stage when surgical intervention rather than preventive management or self-care is required. A key aspect of prevention is the early detection of disease, and prompt management with preventive therapies which increases the beneficial effects of the treatment. Towards this, the focus of health professionals should be improved diagnosis, while public awareness regarding oral diseases and the importance of routine professional evaluation should be increased. Examples of preventive measures for common oral health conditions are presented in Table 3 [32].

Dental professionals must stress the importance of recall visits which are crucial to prevent disease progression. An interval of 2–6 months is considered reasonable depending on professional assessment, and dental professionals must counsel patients on adhering to follow-up visits as well as the necessity of continued care. Considering the systemic implications of oral diseases and the common risk factors with NCDs, dentists must counsel patients on lifestyle changes including smoking and alcohol cessation. In addition, patients should be counseled on the choice of oral products including toothpastes, mouthwashes, etc., and the correct method of using the appropriate products. It is necessary to address the knowledge gap regarding the methods, time and frequency of using evidence-based oral hygiene products. A holistic, interdisciplinary approach by dental professionals and other healthcare providers is crucial to the management of oral diseases.

## 6. Conclusions

Given the increasing prevalence of oral diseases and the link with systemic health, it has become increasingly important to raise public awareness on the importance of oral health, and the role of preventive measures in oral health. Appropriate preventive measures and ideal oral hygiene habits must be adopted at an early age to prevent oral diseases. In addition, people must be encouraged to routinely undergo oral health check-ups to detect oral diseases at early stages wherein management with non-surgical approaches is adequate. In addition, oral health should be a part of the medical curriculum and knowledge of the systemic link with oral diseases should be included in the dental curriculum. Primary care physicians must have knowledge of oral health conditions, enabling them to help patients identify early symptoms of oral diseases, educating patients on oral diseases and referring them to dental professionals for further care. Medical professionals can thus contribute to early detection and prevention of oral diseases during their interaction with patients who may have various risk factors for oral diseases. This would help foster an interprofessional collaborative approach for the management of oral health.

## Figures and Tables

**Table 2:** Risk factors for periodontal diseases [49,50,51,52,53,54,55,56].

Non-Modifiable Risk Factors	Modifiable Risk Factors
Age	Smoking
Gender	Poorly controlled diabetes mellitus
Hormonal variations	Lack of oral hygiene
Some hematological disorders	Obesity
Genetics	Improper diet
	Chronic inflammation
	Drug-induced disorders (including decreased salivation, increased gingival growth, and altered pH and composition of plaque)
	Stress

**Table 3:** Examples of preventive measures for common oral diseases [32].

Oral Disease	Preventive Measures		
	Lifestyle Measure	Self-Care Practices	Professional Care
Periodontal diseases	Reduction in sugar intake Smoking cessation Reduction in alcohol consumption	Tooth brushing Interdental cleaning Use of toothpaste and mouthwashes	Professional mechanical plaque removal (PMPR) Providing oral hygiene instruction
Dental caries	Reduction in sugar intake	Use of fluoride toothpaste Interdental cleaning	Use of fluoride varnish/gel to achieve remineralization Providing oral hygiene instruction
Dentine hypersensitivity	Avoiding erosive food and beverages	Use of a soft-bristled toothbrush Use of toothpaste for sensitivity	Providing oral hygiene instruction

## Data Availability

No new data were created or analyzed in this study. Data sharing is not applicable to this article.
